# Endothelial Dysfunction, Inflammation, and Apoptosis in Diabetes Mellitus

**DOI:** 10.1155/2010/792393

**Published:** 2010-06-15

**Authors:** Inge A. M. van den Oever, Hennie G. Raterman, Mike T. Nurmohamed, Suat Simsek

**Affiliations:** ^1^Department of Rheumatology, Jan van Breemen Institute, Amsterdam, The Netherlands; ^2^Department of Rheumatology, VU University Medical Center, Amsterdam, The Netherlands; ^3^Department of Internal Medicine, VU University Medical Center, Amsterdam, The Netherlands; ^4^Department of Internal Medicine, Medical Center Alkmaar, Wilhelminalaan 12, 1815 JD Alkmaar, The Netherlands

## Abstract

Endothelial dysfunction is regarded as an important factor in the pathogenesis of vascular disease in obesity-related type 2 diabetes. The imbalance in repair and injury (hyperglycemia, hypertension, dyslipidemia) results in microvascular changes, including apoptosis of microvascular cells, ultimately leading to diabetes related complications. This review summarizes the mechanisms by which the interplay between endothelial dysfunction, inflammation, and apoptosis may cause (micro)vascular damage in patients with diabetes mellitus.

## 1. Introduction

The rapidly increasing prevalence of diabetes mellitus worldwide is one of the most serious and challenging health problems in the 21st century.

The number of people with diabetes grows faster than expected. In 2007, 246 million people (roughly 6%) were affected worldwide and it is estimated that this will increase to 380 million, or 7.3% by 2025. Furthermore, it is estimated that there are even more people (308 million or 8.1%) with impaired glucose tolerance (IGT). These people have a significant risk of developing type 2 diabetes mellitus (T2DM).

Diabetes is a metabolic disorder which is characterized by hyperglycemia and glucose intolerance due to insulin deficiency, impaired effectiveness of insulin action or, both.

Type 1 diabetes mellitus (T1DM) is caused by cellular-mediated autoimmune destruction of pancreatic islet beta-cells leading to loss of insulin production. It usually starts during childhood, but can occur at all ages. T2DM accounts for 90%–95% of all diabetes and is more common in people older than 45 who are overweight. There is strong evidence that genetics plays an important role as well. However, the prevalence of T2DM is becoming higher in children and young adults because of the higher rate of obesity in this population.

Central obesity and insulin resistance next to diabetes, high cholesterol and high blood pressure form the most important risk factors for cardiovascular disease (CVD). CVD is the major cause of death in people with T2DM. Diabetes is also the leading cause of blindness, renal failure, and lower limb amputations [1, 2]. 

Dysfunction of the endothelium is regarded as an important factor in the pathogenesis of vascular disease in diabetes mellitus [3–5]. The endothelium is the active inner monolayer of the blood vessels, forming an interface or barrier between circulating blood in the lumen and the rest of the vessel wall, and plays a critical role in vascular homeostasis. It actively regulates vascular tone and permeability, the balance between coagulation and fibrinolysis, the inflammatory activity and cell proliferation. The endothelium even affects the functions of other cell types, such as vascular smooth muscle cells (VSMC's), platelets, leukocytes, retinal pericytes, renal mesangial cells, and macrophages, amongst others through the production of several chemical mediators [3–8]. In health, endothelial cell injury is mitigated by endogenous reparative processes.

An imbalance in repair and injury resulting in early microvascular changes, including apoptosis of microvascular cells, can be seen in both experimental diabetic animal models and humans with diabetes. Several studies indicate that microvascular cell apoptosis plays an important role in the development of early lesions [6, 8, 9]. 

We will review the role of endothelial dysfunction and especially inflammation-induced apoptosis of endothelial cells in obesity-related diabetes mellitus and its co-morbidities.

## 2. Endothelial Function and Dysfunction

**Figure 1:**
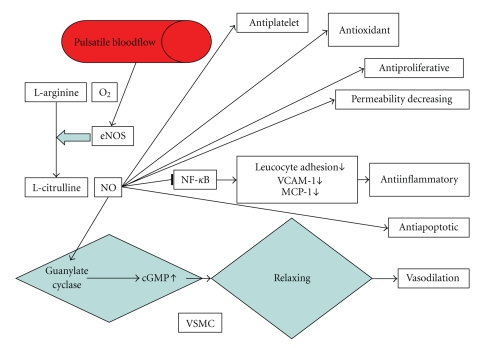
Properties and production process of NO (nitric oxide) as important factor in endothelial function.

To maintain vascular homeostasis, the endothelium produces components of the extracellular matrix such as collagen and a variety of regulatory chemical mediators, including nitric oxide (NO), prostanoids (prostacycline), endothelin-1 (ET-1), angiotensin II (ANG-II), tissue-type plasminogen activator (t-PA), plasminogen activator inhibitor-1 (PAI-1), von Willebrand factor (vWF), adhesion molecules (VCAM, LAM, ICAM), and cytokines, among them Tumor Necrosis Factor *α* (TNF*α*) [10] (Figure 1).

The endothelium has a prominent role in maintaining blood fluidity and restoration of vessel wall integrity to avoid bleeding. It regulates fibrinolysis by producing t-PA and its inhibitor PAI-1 and limits activation of the coagulation cascade by thrombomodulin/protein C, heparin sulphate/antithrombin and tissue factor/tissue factor inhibitor interactions. Through release of promoters and inhibitors of growth and differentiation of the VSMC, such as platelet-derived growth factor (PDGF) and ANG-II, endothelium also has an impact on vascular remodeling [11]. 

ANG-II exerts regulatory effects on several VSMC activities including contraction, growth, proliferation, and differentiation. By the production of adhesion molecules like leukocyte adhesion molecule (LAM), intracellular adhesion molecule (ICAM), and vascular cell adhesion molecule (VCAM), inflammatory cells are attracted and anchored, thereby playing a regulatory inflammatory role [12, 13]. 

Endothelial dysfunction is the change of these properties, either in the basal state or after stimulation, that is inappropriate with regard to the preservation of organ function. The kind of changes that occur, can depend on the type of injury and may depend on the intrinsic properties of the endothelium (venous versus arterial endothelium).

Under physiological circumstances, there is a balanced release of endothelial-derived relaxing factors such as nitric oxide (NO) and prostacyclin (PGI2), and contracting factors such as endothelin-1 (ET-1), prostaglandins, and angiotensin II (ANG-II). In endothelial dysfunction, this balance is altered, predisposing the onset and progression of atherosclerosis [14]. Risk factors such as hypercholesterolemia, dyslipidemia, smoking, and diabetes initiate atherosclerosis through endothelial activation and therefore through endothelial dysfunction. Endothelial dysfunction is expressed in increased interactions with leukocytes, smooth muscle growth, vasoconstriction, impaired coagulation, vascular inflammation, thrombosis, and atherosclerosis [15].

A very important mediator synthesized by endothelial cells is nitric oxide (NO), because of its vasodilatory, antiplatelet, antiproliferative, permeability-decreasing, antiinflammatory, and antioxidant properties [16]. NO inhibits rolling and adhesion of leucocytes as well as cytokine-induced expression of vascular cell adhesion molecule-1 (VCAM-1) and monocyte chemotactic protein-1 (MCP-1) [17], probably through the inhibition of the transcription factor nuclear factor *κ* B (NF-*κ*B) [14, 18, 19].

NO is produced through the conversion of the amino acid l-arginine to l-citrulline by the enzyme NO-synthase (NOS). There are several isoforms: NOS1 isolated from the brain, NOS2, or iNOS, produced by macrophages and NOS3 or eNOS from endothelial cells. eNOS is activated by the pulsatile flow of blood through vessels. eNOS produces NO which diffuses to the vascular smooth muscle (VSM) where it activates the enzyme guanylate cyclase which in turn increases cyclic GMP and thereby induces relaxation of the VSM. In this way it maintains the diameter of the blood vessel ensuring optimal perfusion of tissues. NOS is regulated by bradykinin, which acts with b2 receptors on the endothelial cell surface membrane, increasing the production of NO via NOS activation. The local concentrations of bradykinin are regulated by the activity of angiotensin converting enzyme (ACE), by breaking down bradykinin into inactive peptides [20, 21]. 

Endothelial dysfunction is associated with decreased NO availability, either through loss of NO production or through loss of NO biological activity [22]. NO production is diminished in cells which are subject to oxidative stress. Oxidative stress is caused by three factors: (1) an increase in oxidant generation, (2) a decrease in antioxidant protection, (3) a failure to repair oxidative damage. Cell damage is induced by reactive oxygen species (ROS), which are either free radicals, reactive anions containing oxygen atoms, or molecules containing oxygen atoms that can either produce free radicals or are chemically activated by them. Examples are hydroxyl radical, super oxide, hydrogen peroxide, and peroxynitrite. Normally these ROS are scavenged by different intra- and extra cellular mechanisms, but in a situation of oxidative stress these mechanisms are insufficient to cope with the exaggerated generation of ROS. NO may react with some ROS species to form peroxynitrite, in turn increasing the oxidative stress in the cell. Several cardiovascular risk factors like hyperglycemia, insulin resistance, dyslipidemia, inflammation, and also cigarette smoking may induce oxidative stress [5, 19]. 

Oxidative stress is an important factor which can induce cell apoptosis. In the next part, we will explain the process of apoptosis.

## 3. Apoptosis

Apoptosis is the process in which a cell plays an active role in its own death. This is why it is also called cell suicide. Apoptosis differs from necrosis in the level of control of the process. Apoptosis is a controlled and regulated process and involves individual cells. Necrosis is an uncontrolled process of cell lysis leading to inflammation and destruction of tissue areas or even whole organs, which can cause serious health problems. Apoptosis, or programmed cell death, is a normal component of the development and health of multicellular organisms and continues throughout adult life. Apoptosis and proliferation are responsible for shaping tissues and organs in developing embryos. During adult life, apoptosis is a protection mechanism which eliminates old, useless, and damaged cells. In healthy organisms apoptosis and cell proliferation are in balance. In diseases such as cancer there is an imbalance whereby cells have undergone certain mutations that prevent them from undergoing apoptosis. In neurodegenerative diseases such as Parkinson's disease apoptosis is thought to account for the excessive loss of neurons.

There are several mechanisms through which apoptosis can be induced in cells. There are extrinsic signals such as the binding of death inducing ligands to cell surface receptors also called death receptors. Some of these ligands are expressed on the surface of cytotoxic T lymphocytes, for example, when a cell is infected by a virus. Apoptosis can also be induced by intrinsic signals, that are produced following cellular stress. Cellular stress can be caused by oxidative stress through free radicals, deprivation of growth factor, or exposure to radiation or chemicals. The sensitivity of cells to these stimuli can vary depending on a number of factors, such as the expression of pro- and antiapoptotic proteins, the severity of the stimulus and the stage of the cell cycle.

Very important death inducing ligands are the Fas ligand, TNF*α* and TRAIL (TNF related apoptosis inducing ligand). When they bind their specific death receptor, apoptotic signals are transmitted in the cell and a caspase cascade is activated within seconds of ligand binding, inducing apoptosis in a very rapid way. The general signaling pathway that is activated through death receptor binding begins with the generation of ceramide, produced by acid sphingomyelinase. Ceramide release promotes lipid raft fusion which results in clustering of death receptors. This is important because it helps amplify the apoptotic signaling. A conformational change in the intracellular domains of the death receptors reveals the presence of a death domain which allows the recruitment of various apoptotic proteins to the receptor. This is called the death inducing signaling complex (DISC). As a final step, the DISC recruits and activates procaspase 8. Caspase 8 initiates the apoptosis of the cell.

The sensitivity of cells to apoptotic stimuli can depend on the balance of pro- and antiapoptotic bcl-2 proteins. Bcl-2 and bcl-XL are antiapoptotic, while Bad, Bax and Bid are proapoptotic proteins [23, 24]. The proapoptotic bcl-2 proteins are often found in the cytosol acting as sensors of cellular damage or stress. In case of cell stress they relocate to the surface of mitochondria where the antiapoptotic proteins are located. This interaction between pro- and antiapoptotic proteins leads to the formation of Permeability Transition pores (PTP) in the mitochondrial membranes [25]. Recent evidence implies that there may also be a mitochondrial apoptotic pathway distinct from that activated by proaptotic bcl-2 family proteins, dependent on cyclophilin D [26]. The mitochondria contains proapoptotic proteins such as Apoptosis Inducing Factor (AIF), Smac/DIABLO, and cytochrome C, which are released through these pores, which in turn leads to the formation of the apoptosome and the activation of the caspase cascade [27, 28].

Once cytochrome C is released into the cytosol, it interacts with apoptotic peptidase activating factor-1 (APAF-1) and this leads to the recruitment of procaspase 9 into a multiprotein complex called the apoptosome. Activation of caspase 9 through formation of the apoptosome causes apoptosis.

Nitric oxide has been demonstrated to inhibit apoptosis in a number of cell types including endothelial cells. The antiapoptotic effects can be mediated through mechanisms such as nitrosylation and inactivation of caspase 1, 3 and 8. Other mechanisms include activating p53, upregulating heat shock protein 70, and upregulating antiapoptotic proteins Bcl-2 and Bcl-XL. Through activation of cGMP signaling, caspase activity is suppressed, cGMP-dependent protein kinases are activated and possibly the expression of antiapoptotic proteins increases. Apoptosis and especially apoptosis of endothelial cells may be highly significant in the development of diabetes and atherosclerosis [29]. 

## 4. Endothelial Cell Dysfunction and Apoptosis in Diabetes

Dysfunction of endothelium in diabetes mellitus is characterized by changes in proliferation, barrier function, adhesion of other circulating cells, and sensitivity to apoptosis. Furthermore, it is suggested that diabetes mellitus modifies angiogenic and synthetic properties of endothelial cells [30–36]. 

There is a lot of evidence that endothelial dysfunction is closely connected to the development of diabetic retinopathy, nephropathy, and atherosclerosis in both T1DM and T2DM [4, 37]. But, what are the specific mechanisms that cause this close association between diabetes and endothelial dysfunction? Large clinical trials in both T1DM and T2DM have shown that hyperglycemia plays a big part in the pathogenesis of microvascular complications and is a major causal factor in the development of endothelial dysfunction and endothelial cell apoptosis [5, 38, 39]. However, the exact mechanism of hyperglycemia-related tissue damage and clinical complications remains unclear. There is also a significant role for insulin and especially insulin resistance, as increasing evidence implies that the obesity-related progression of insulin resistance to T2DM parallels the progression of endothelial dysfunction to atherosclerosis. Still this relationship has been difficult to prove because insulin resistance is often accompanied by a cluster of other risk factors as mentioned above.

## 5. Endothelial Dysfunction and Apoptosis in T2DM

The role of endothelial dysfunction in T2DM is very complicated, due to the many independent factors involved, including ageing, obesity, hyperlipidemia, hypertension, low grade inflammation, insulin resistance, and hyperglycemia [40]. All of these factors are associated with the metabolic syndrome, which usually precedes T2DM. The relationship of endothelial dysfunction and all of these factors is not completely understood despite extensive research. Even the question whether endothelial dysfunction is a consequence or the cause of all the changes occurring in the metabolic syndrome and diabetes cannot be answered easily. In the next few paragraphs we will discuss the relation between endothelial dysfunction and the individual factors mentioned above, starting with insulin resistance.

### 5.1. Insulin Resistance

**Figure 2:**
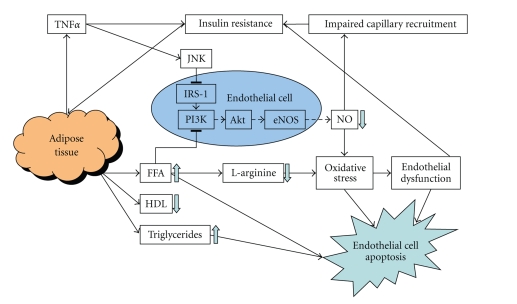
Mechanisms of insulin resistance and adipose tissue in relation to endothelial dysfunction and apoptosis.

Insulin resistance is defined as the decreased ability of insulin to promote glucose uptake in skeletal muscle and adipose tissue and the decreased hepatic output of glucose. This may be present years before the development of abnormal plasma glucose levels becomes evident [41, 42] (Figure 2). 

Insulin resistance is associated with an increased free fatty acids (FFA) release from adipose tissue, which results in dyslipidemia, including VLDL-hypertriglyceridemia, high plasma FFA, and low HDL-cholesterol concentrations. High FFA levels and hypertriglyceridemia are associated with endothelial dysfunction. FFA-mediated endothelial dysfunction is probably caused by reduced availability of L-arginine and/or NO and oxidative stress [43]. It has been proven that increased saturated and polyunsaturated FFA concentrations, except for oleic acid, directly induce cell cycle arrest and apoptosis in vascular endothelial cells [44]. 

Insulin is a vasoactive hormone and enhances muscle blood flow and vasodilation via stimulation of NO production. The increased blood flow caused by insulin, differs among different types of vessels. Insulin can also redirect blood flow in skeletal muscles so that more glucose can be uptaken by muscle cells. This process is called capillary recruitment. In T2DM, hypertension and obesity, insulin's vasodilator actions are impaired, probably for a large part because of low NO action. Normally, stimulation of NO production by insulin is mediated by signaling pathways involving activation of Phosphoinositide-3 (PI-3) kinase leading to phosphorylation of eNOS. It is suggested that endothelial dysfunction and impaired capillary recruitment can cause insulin resistance because the microvascular endothelium can not react properly to insulin and glucose disposal is decreased. This is called endothelial insulin resistance. How metabolic and endothelial insulin resistance originate and their exact relationship are not fully understood. Both TNF*α* and nonesterified acids (NEFAs) can cause metabolic and endothelial insulin resistance. Inflammatory cytokines like TNF*α*, can act as mediators of insulin resistance by impairing the tyrosine kinase activity of both the insulin receptor (IR) and insulin receptor substrate (IRS-1), thus inhibiting insulin signaling. It is suggested that a bidirectional relationship exists between hyperinsulinemia and low-grade chronic inflammation, by which hyperinsulinemia can lead to vascular inflammation and vascular inflammation causes insulin resistance and finally compensatory hyperinsulinemia. At normal physiological concentrations insulin exerts prevailing antiinflammatory effects, while hyperinsulinemia increases levels of oxidative stress and inflammation. A recent study with Human Umbilical Vein Endothelial Cells (HUVECs) shows that insulin, at pathophysiological concentrations alone or in combination with low concentrations of TNF*α*, has the ability to promote VCAM-1 expression, through increasing the steady state levels of mRNA via the activation of transcription factors, such as NF-*κ*B, which has been linked to VCAM-1 transactivation before. This way, hyperinsulinemia leads to increased monocytoid cell adhesion to HUVECs [5, 19, 45]. 

A very important effect of insulin resistance is the fact that the normal route for insulin to activate the PI-3 kinase and Akt-dependent signaling pathways is impaired, whereas hyperinsulinemia overactivates Mitogen activated protein kinases (MAPK)-pathways, thereby creating an imbalance between PI-3 kinase and MAP-kinase-dependent functions of insulin. This probably leads to decreased NO production and increased ET-1 secretion, characteristic of endothelial dysfunction. Through activation of the MAP-kinase signaling pathways, hyperinsulinemia promotes secretion of ET-1, activates cation pumps, and increases expression of VCAM-1 and E-selectin [46]. ET-1, a vasoconstrictor, can increase serine phosphorylation of IRS-1, causing a decreased activity of PI-3 kinase in vascular smooth muscle cells. Moreover, ET-1 may also impair insulin-stimulated translocation of GLUT-4 in adipocytes [47, 48]. 

### 5.2. Hypertension

Hypertension induces endothelial activation and probably also endothelial dysfunction and is a major determinant of microangiopathy and atherothrombosis in diabetes. Hypertension is associated with insulin resistance and this relation can partly be explained by decreased capillary density and impaired capillary recruitment seen in insulin resistant states. Another explanation is the fact that NO availability is diminished and ET-1 availability is increased in both insulin resistance and hypertension. The exact link between diabetes and hypertension is not fully known [49]. 

### 5.3. Obesity

The adipose tissue has become known to be a highly active endocrine organ, releasing hormones, cytokines, and enzymes with the tendency to impair insulin sensitivity. It is an important modulator of endothelial function via secretion of a variety of hormones, including adiponectin, resistin, leptin, PAI-1, angiotensin, estradiol, and the cytokines TNF*α* and interleukin-6 (IL-6). Plasma adiponectin levels are reduced in people with obesity and also in people with diseases associated with obesity, like T2DM and coronary artery disease. Adiponectin has antiinflammatory features and is inversely related to BMI, oxidized LDL, insulin resistance, and atherosclerosis [19]. It plays an important role in fatty acid metabolism and glucose homeostasis. Low adiponectin levels are associated with an increased oxidative state in the arterial wall and systemic oxidative stress. In endothelial cells, adiponectin increases the production of nitric oxide and suppresses oxidative stress and the inflammatory signaling cascades via AMP-activated protein kinases (AMPK) and the cyclic AMP-protein kinase A-linked pathway [50]. Moreover, it reduces the attachment of monocytes to endothelial cells and inhibits the expression of adhesion molecules [5, 51]. 

The role of resistin in insulin resistance and diabetes is controversial since a number of studies have shown that resistin levels increase with increased central adiposity and other studies have demonstrated a significant decrease in resistin levels in increased adiposity. PAI-1 is present in increased levels in obesity and the metabolic syndrome. It has been linked to the increased occurrence of thrombosis in patients with these conditions.

Angiotensin II is also present in adipose tissue and has an important effect on endothelial function. When angiotensin II binds the angiotensin II type 1 receptor on endothelial cells, it stimulates the production of ROS via NADPH oxidase, increases expression of ICAM-1 and increases ET-1 release from the endothelium [52–54]. Angiotensin also activates JNK and MAPK pathways in endothelial cells, which leads to increased serine phosphorylation of IRS-1, impaired PI-3 kinase activity and finally endothelial dysfunction and probably apoptosis. This is one of the explanations why an ACE inhibitor and angiotensin II type 1 receptor blockers (ARBs) protect against cardiovascular comorbidity in patients with diabetes and vice versa [55]. 

Insulin receptor substrate 1 (IRS-1) is a protein downstream of the insulin receptor, which is important for signaling to metabolic effects like glucose uptake in fat cells and NO-production in endothelial cells. IRS-1 in endothelial cells and fat cells can be downregulated by stressors like hyperglycemia and dyslipidemia, causing insulin resistance and endothelial dysfunction. A low adipocyte IRS-1 expression may thereby be a marker for insulin resistance [19, 56, 57].

### 5.4. Inflammation

Nowadays atherosclerosis is considered to be an inflammatory disease and the fact that atherosclerosis and resulting cardiovascular disease is more prevalent in patients with chronic inflammatory diseases like rheumatoid arthritis, systemic lupus erythematosus and ankylosing spondylitis than in the healthy population supports this statement. Inflammation is regarded as an important independent cardiovascular risk factor and is associated with endothelial dysfunction.

Interestingly, a study performed by bij van Eijk et al. shows that patients with active ankylosing spondylitis, an inflammatory disease, also have impaired microvascular endothelium-dependent vasodilatation and capillary recruitment in skin, which improves after TNF*α*-blocking therapy with etanercept [58].

The existence of chronic inflammation in diabetes is mainly based on the increased plasma concentrations of C-reactive protein (CRP), fibrinogen, interleukin-6 (IL-6), interleukin-1 (IL-1), and TNF*α* [59–61]. Inflammatory cytokines increase vascular permeability, change vasoregulatory responses, increase leukocyte adhesion to endothelium, and facilitate thrombus formation by inducing pro-coagulant activity, inhibiting anticoagulant pathways and impairing fibrinolysis via stimulation of PAI-1. NF-*κ*B consists of a family of transcription factors, which regulate the inflammatory response of vascular cells, by transcription of various cytokines which causes an increased adhesion of monocytes, neutrophils, and macrophages, resulting in cell damage. On the other hand, NF-*κ*B is also a regulator of genes that control cell proliferation and cell survival and protects against apoptosis, amongst others by activating the antioxidant enzyme superoxide dismutase (SOD) [62]. NF-*κ*B is activated by TNF*α* and IL-1 next to hyperglycemia, AGEs, ANG-II, oxidized lipids, and insulin. Once activated, NF-*κ*B translocates from the cytoplasm to the nucleus to activate gene transcription. NF-*κ*B-regulated genes are VCAM-1, E-selectin, ICAM-1, IL-1, IL-6, IL-8, tissue factor, PAI-1, and NOS.

The TNF-family of cytokines plays an important role in regulating the immune response, inflammation, and apoptosis. The first cytokine discovered is TNF*α*, which is produced by neutrophils, macrophages, and adipocytes and can induce other powerful cytokines such as IL-6, which in turn regulates the expression of C-reactive protein (CRP). CRP increases the expression of endothelial ICAM-1, VCAM-1, E-selectin, MCP-1 and increases the secretion of ET1. Moreover, CRP decreases eNOS expression and elevates the expression of angiotensin receptor type 1 in the vessel wall [63, 64].

TNF*α* can induce insulin resistance and this is probably a part of the explanation why insulin resistance, endothelial dysfunction, and atherothrombosis are so closely related. Recent studies indicate that TNF*α* is likely involved in the pathogenesis of diabetic nephropathy and retinopathy. A very recent study with T1DM and T2DM rats shows that TNF*α* plays an important role in microvascular apoptosis in diabetes. When the diabetic rats were treated with pegsunercept, a TNF*α* inhibitor, a significant reduction of the number of endothelial cells that expressed activated caspase-3 by 76% to 80% occurred. TNF*α* inhibition decreases intercellular adhesion molecule 1 (ICAM-1) levels and NF-*κ*B activity in diabetic retina. Another study in diabetic rats demonstrated that increased levels of TNF*α* consequently enhanced FOXO-1 mRNA levels, nuclear translocation, and DNA binding in retinas of T1DM and T2DM rats. It also showed that the transcription factor FOXO-1, which regulates cell death; prevents cell cycle progression, modulates differentiation in various cell types, plays a critical role in diabetes-induced apoptosis and retinal microvascular cell loss [65]. It is possible that TNF*α* upregulation may contribute to increased apoptosis detected in other diabetes associated complications and TNF*α* inhibition may be a potential therapeutic option in preventing this comorbidity [66]. 

Tumor necrosis factor alpha-Related Apoptosis-Inducing Ligand (TRAIL), also known as APO2L, is another member of the TNF family of cytokines and is a type II membrane protein. The effects induced by TRAIL are mediated by interactions with cell surface TRAIL receptors. Five TRAIL receptors have been found so far in humans. When TRAIL binds TRAIL-R1 (DR4) and TRAIL-R2 (DR5) apoptotic signals are transduced. TRAIL-R3 (DcR1), TRAIL-R4 (DcR2), and osteoprogeterin (OPG) lack an intracellular death domain and can not induce apoptosis. Uniquely, TRAIL can exert anticancer activity, while causing no or minimal organ toxicity and inflammation. TRAIL acts among others on nuclear factor kappa B (NF-*κ*B). TRAIL induces the release of NO by vascular endothelial cells [67]. Studies have shown that OPG is remarkably increased in diabetic patients and even more so in patients with cardiovascular disease, like coronary artery disease or abdominal aortic aneurysm [68, 69]. In a study with SZT-induced rats and a control group of healthy rats the OPG/TRAIL ratio was markedly increased in the diabetic animals with respect to the control animals. The next remarkable observation in this study was the ability of insulin to downregulate TRAIL expression in rat aortas in vivo.

Further investigation of the role of insulin in the TRAIL expression in diabetes was done with VSMCs in vitro. This showed the same result: a decrease of surface TRAIL expression. High glucose levels did not show any significant effect on TRAIL surface expression in both studies. These findings suggest that the downregulation of TRAIL expression may play a role in diabetic vasculopathy. A possible explanation for these results is the upregulation of the transcription factor early growth response protein 1 (Egr-1), which in turn downregulates TRAIL expression in endothelial cells, by both hyperglycemia and insulin. A supportive finding for this hypothesis is the fact that VEGF receptor 1 (FLT1) and PAI-1, both known Egr-1 responsive genes, are also increased in the presence of glucose and insulin. Thus, Egr-1 upregulation, which is frequently observed in atherosclerosis, is likely to be involved in insulin-mediated TRAIL downregulation [70]. 

Plasma levels of C-reactive protein (CRP) are increased in both T1DM and T2DM. CRP plays a significant role in atherogenesis in endothelial cells, next to vascular smooth muscle cells and macrophages, and several studies have revealed that CRP levels predict cardiovascular disease [71]. CRP causes numerous proinflammatory and pro-atherogenic effects in endothelial cells, such as decreased NO and prostacyclin, increased ET-1, cell adhesion molecules, MCP-1, IL-8, and PAI-1 [5]. 

Another important contribution to chronic inflammation in diabetes is caused by primed peripheral polymorphonuclear leukocytes (PMNs). In a small study with T2DM patients and a control group, it was shown that T2DM patients are exposed to oxidative stress and chronic inflammation partially because of the primed state of their PMNs, amongst others because these primed PMNs release superoxide significantly faster than normal control PMNs. Apoptosis in primed PMNs was also higher in the diabetic patients, probably partly because of intracellular factors such as high cytosolic calcium concentrations [72]. At the same time apoptosis of normal PMNs of the control group was significantly higher in diabetic serum, suggesting leucoclastic activity of diabetic serum. This was confirmed by the findings of Abu El-Asrar et al. [73]. This study also observed a decrease in plasma gluthathione (GSH), an intra- and extra cellular antioxidant, which neutralizes oxidants, including hydrogen peroxide and superoxide, by converting them to other oxidized forms [61].

### 5.5. Dyslipidemia

Dyslipidemia is characterized by low HDL-cholesterol levels and an excess of small, dense LDL and is associated with obesity, insulin resistance and diabetes in general. An increase in postprandial triacylglycerol-rich lipoproteins, like chilomicrons and -LDL particles, enhances oxidative stress and consequently causes endothelial dysfunction and increased apoptosis [74]. 

## 6. Hyperglycemia and Endothelial Dysfunction

**Figure 3:**
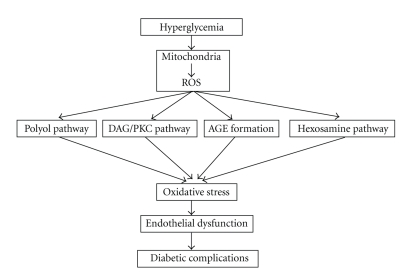
Mechanisms of hyperglycemia which are supposed to cause endothelial dysfunction and in the end diabetic complications.

There have been various mechanisms discovered that can explain how hyperglycemia causes vascular complications. There are several pathways which get activated through hyperglycemia and can potentiate each other. The basis for the activation of these pathways is most likely the overproduction of ROS in mitochondria induced by hyperglycemia (Figure 3).

### 6.1. The Polyol/Sorbitol/Aldose Reductase Pathway

In a lot of cells excess glucose is reduced to sorbitol by aldose reductase. Sorbitol is later metabolized to fructose by sorbitol dehydrogenase, the polyol pathway. At the same time it increases the oxidation of NADPH to NADP+ and the reduction of NAD+ to NADH, the co-factors, which in turn decreases NO bioavailability [75]. This causes a redox imbalance that resembles tissue hypoxia and is therefore called hyperglycemic pseudohypoxia. It also increases the formation of methylglyoxal and AGEs. All these processes enhance oxidative stress [76]. The increased sorbitol accumulation increases osmotic stress and decreases other osmolytes such as myo-inositol and taurine. A study in rat and human retinas produced evidence that the polyol pathway may have an important role in diabetic retinopathy. It also proved that the aldose reductase inhibitor (sorbinol) prevents vascular processes, culminating in the development of acellular capillaries [5, 75, 77]. This may imply that the polyol pathway can cause endothelial cell apoptosis. However, the full impact of this pathway in the endothelial dysfunction is not completely understood yet.

### 6.2. The DAG/PKC Pathway

The hyperglycemia induced activation of the diacylglycerol (DAG)-protein kinase C (PKC) pathway has multiple adverse effects on the vascular function. Hyperglycemia increases the levels of DAG, which in turn activates PKC. In hyperglycemic circumstances DAG is synthesized from the glycolytic intermediates dihydroxyacetone phosphate (DHAP) and glycerylaldehyde-3-phosphate, by a de novo pathway [78]. Oxidants like H_2_O_2_ can also activate the DAG/PKC pathway. There are at least eleven PKC isoforms. In vascular cells the isoform PKC-beta-II is most frequently activated [79]. The pathogenic consequences of PKC activation include dysregulation of the vascular permeability through the induction of vascular endothelial growth factor (VEGF) in smooth muscle cells [80], dysregulation of blood flow by decreasing endothelial NOS activity and/or increasing ET-1 synthesis [81], basement membrane thickening through Transforming Growth Factor-beta (TGF-*β*)-mediated increased production of type IV collagen and fibronectin, increased expression of PAI-1 which causes impaired fibrinolysis and activation of superoxide producing enzymes like NADPH as well as an increased expression of a dysfunctional, superoxide-producing, uncoupled endothelial NOS, thus increasing oxidative stress [5]. 

Recently, Geraldes et al. have identified a new signaling pathway by which hyperglycemia causes increased vascular cell pathology and apoptosis resulting in diabetic retinopathy in mouse retinas. They proved that hyperglycemia, especially in pericytes, activates PKC-*δ*, probably through an increase in transcription of the gene encoding PKC-*δ*. This as well as activation of p38*α* MAPK leads to increased expression of Scr homology-2 domain-containing phosphatase-1 (SHP-1), which subsequently induces apoptosis via deactivation of platelet-derived growth factor *β* (PDGF-*β*) [82].

### 6.3. Non-Enzymatic Glycation End Products (AGE)

Non-enzymatic glycation products are a complex and heterogeneous group of compounds which accumulate in plasma and tissues in diabetes and renal failure. There is emerging evidence that these compounds play a role in the pathogenesis of chronic complications associated with diabetes and renal failure. Earlier research in both diabetic animals and humans revealed an association between the accumulation of AGE-modified proteins and the severity of microvascular complications. The second evidence stems from the fact that typical microvascular complications develop following injections of AGE-modified proteins in non-diabetic animals [83].

The advanced glycation end-products (AGE) concept proposes that chemical modification and cross linking of tissue proteins, lipids, and DNA affect their structure, function and turnover, contributing to a gradual decline in tissue function and to the pathogenesis of diabetic complications. Nonenzymatic glycation of proteins is a condensation reaction between the carbonyl group of free glucose and the N-terminus of reactive-protein amino groups, like lysine or arginine, yielding Schiff-base intermediates that undergo Amadori rearrangement to form stable proteinglucose adducts, for example glycated hemoglobin A1c (HbA1c) and fructosamine (fructoselysine). Amadori-modified matrix proteins are increased in diabetes. Because Amadori-adducts are relatively stable, only a small fraction undergoes rearrangements to irreversible AGEs. At first it was believed that AGEs are only formed on long-lived extra cellular molecules, because of the slow rate of reaction of glucose with proteins. However, other sugars like glucose-6-phosphate and glyceraldehyde-3-phophate can also create AGEs with intracellular and short-lived molecules and at a much faster rate than glucose. AGEs can arise from the decomposition of Amadori products, from fragmentation products of the polyol pathway, and as glycoxidative products which all react with protein amino groups. When oxidation is involved, the so-called glycoxidation products such as pentosidine and carboxymethyllysine are formed. It has recently been found that glucose can probably autoxidize to form reactive carbonyl compounds (glyoxal, methylglyoxal and 3-deoxyglucosone) which may react with protein to form glycoxidation products. In endothelial cells methylglyoxal is probably the main AGE formed. AGEs can interfere with the endothelial function in several ways. They can act as oxidants and cause generation of reactive oxygen species (ROS). AGEs can decrease arterial elasticy and AGE modified type I and IV collagen can prevent normal matrix formation and cross-linking. Interactions of mononuclear cells and macromolecules like LDL with the endothelial wall are stimulated by AGE-modified matrix, through increased expression of endothelial adhesion molecules. AGEs can also impair the binding of heparan sulfate to the extra cellular matrix, which results in a loss of anionic sites and thus in an increase in endothelial permeability. Early diabetic micro angiopathy is characterized by vasodilation, increased blood flow, and increased capillary permeability. AGE-modified proteins may lead to all these changes.

When AGEs get into the blood circulation they are highly reactive but are often detoxified by various enzymes. When they are not eliminated by the kidneys, recirculating AGE peptides can generate new AGEs reacting with plasma or tissue components. At this stage glycation accelerates the progress of deterioration. Age-modified plasma proteins can bind to AGE receptors (RAGE = AGE-receptor, macrophage scavenger receptor A) on different cell types like endothelial cells, where it can adversely affect the expression of thrombomodulin, tissue factor, and VCAM-1 genes. RAGE-binding mediates signal transduction via a receptor-mediated induction of ROS and activation of transcription factors NF-*κ*B and p21-ras, leading to apoptosis [84]. 

The nonenzymatic glycation of LDL (gLDL) and its role in the pathogenesis of atherosclerosis is a popular subject in studies of late. Due to hyperglycemia, LDL glycation is increased in diabetic patients, however nonenzymatic glycation of LDL happens naturally in all individuals. The modification of LDL by glycation leads to a decreased recognition of LDL by the LDL receptor (LDL-R) and in turn increases the relative circulation time of the lipoprotein, which may result in increased particle oxidation, the formation of AGEs, and the activation of alternative uptake mechanisms by non—LDL-R—mediated pathways. Additionally, gLDL prevents shear stress-mediated L-arginine uptake and nitric oxide formation and causes increased production of plasminogen-activator inhibitor 1 and prostaglandins, while inhibiting the expression of tissue plasminogen activator in endothelial cells [85–87]. Finally gLDL reduces proliferation and triggers apoptosis in HUVECs [44].

It has been proposed that these processes could contribute to the increased susceptibility of diabetic patients to atherosclerosis and coronary heart disease.

So measurement of the products of nonenzymatic glycation has a two-fold meaning: on one hand, measurement of early glycation products can estimate the extent of exposure to glucose and the subjects of previous metabolic control; on the other hand, measurement of intermediate and late products of the glycation reaction is a precious instrument in verifying the relationship between glycation products and tissue modifications.

### 6.4. Hyperglycemia and Oxidative Stress

A single unifying mechanism of the above mentioned pathways has recently been found. The increased production of superoxide anion radicals by mitochondrial electron transport chain plays a key role in the activation of the above pathways. Hyperglycemia-induced superoxide overproduction inhibits GADPH activity by 66%, which is a consequence of poly ADP-ribosylation of GADPH by poly ADP-ribose polymerase (PARP), which in turn is activated by DNA strand-breaks synthesized by mitochondrial superoxide overproduction. This overproduction particularly happens in mitochondria that have been uncoupled by the flux of NADH from the hyperglycemia-enhanced glycolysis. GADPH inhibition causes accumulation of glycolysis intermediates. In aortic endothelial cells, the hyperglycemia induced increased mitochondrial superoxide production and prevented eNOS activity and expression [88]. In addition to mitochondrial uncoupling there are other mechanisms that can contribute to superoxide production in diabetes, namely, uncoupling of eNOS, increased peroxidation and glycoxidation, activation of NADPH oxidases, decreased clearance of superoxide, and impaired antioxidant status [61]. Increased production of ROS causes oxidative stress. Oxidative stress is probably a key event in endothelial dysfunction since inhibition of hyperglycemia, induced, ROS production prevents activation of the aldose reductase, hexosamine pathways, PKC activation, and AGE formation [77, 89]. ROS at low concentrations can function as signaling molecules and participate as signaling intermediates in the regulation of fundamental cell activities, such as cell growth and cell adaptation responses. At higher concentrations they can cause oxidative stress, cellular injury, and apoptosis [7, 90]. ROS can effect many signaling pathways, including G-proteins, protein kinases, ion channels and transcription factors. Finally ROS can modify endothelial function by a variety of mechanisms, like peroxidation of membrane lipids, activation of NF-*κ*B, and decreasing the availability of NO [91]. A recently published study showed that transient exposure of cultured human aortic endothelial to hyperglycemia induces persistent epigenetic changes in the promoter of the NF-*κ*B p65 subunit. In the proximal promoter region of p65, increased monomethylation of histone 3 lysine 4 by the histone methyltransferase Set 7 caused a continuing increase in p65 gene expression, leading to a sustained increase in the expression of the NF-*κ*B-responsive proatherogenic genes MCP-1 and VCAM-1. The cause of these changes was found in the increased generation of methylglyoxal and hyperglycemic-induced ROS formation by the mitochondrial electron transport chain. This means that transient hyperglycemia can cause persistent atherogenic effects during normoglycemia by inducing long lasting chromatin remodeling and vascular epigenetic changes. These results provide a molecular basis for better understanding of the variation in risk for diabetic complications, which can not be explained by HbA1c [92]. 

Oxidative stress is known to induce senescence prematurely in fibroblasts. Cellular senescence or cellular ageing is the phenomenon where normal diploid differentiated cells lose the ability to divide. This phenomenon is also known as “replicative senescence” or the “Hayflick phenomenon”. In response to DNA damage (including shortened telomeres) cells either age or go into apoptosis if the damage cannot be repaired. There is strong evidence as mentioned above, that oxidative stress is increased in diabetic patients. Other studies have revealed that endothelial cells in atherosclerotic lesions show features of cellular senescence, like senescence associated *β*-galactosidase (SA-*β*-gal) staining and telomere shortening. Expression of inflammatory cytokines and adhesion molecules is upregulated in senescent endothelial cells. Furthermore, nitric oxide production is significantly reduced in these cells. More importantly, senescence enhances vascular inflammation and thrombosis in vessels, promoting the development of cardiovascular events. There is also evidence that senescence is more accelerated in patients with diabetes compared to healthy individuals. One study demonstrated that high glucose induced premature cellular senescence in HUVECs through the activation of the Apoptosis Signal-Regulating Kinase 1 (ASK1). Activation of ASK-1 also upregulated PAI-1 expression in the HUVECs and this plus senescence was also observed in aortas of STZ-diabetic wild type mice, whereas this was not seen in STZ-diabetic ASK-1 knock-out mice. PAI-1 is known to play an important role in the pathogenesis of atherosclerosis and thrombosis [93].

## 7. Hyperglycemia and Apoptosis

**Figure 4:**
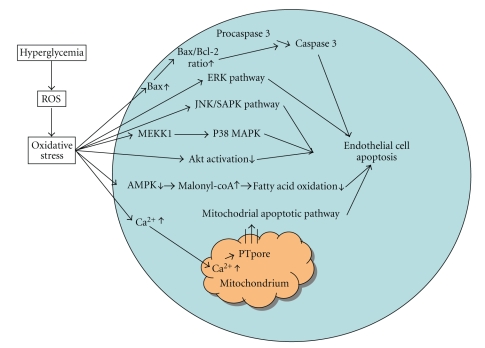
Mechanisms by which hyperglycemia is supposed to induce endothelial cell apoptosis.

The number of (in vitro) studies delivering evidence that hyperglycemia can induce endothelial cell apoptosis [30, 90, 94] has increased extensively over the last few years. These studies have focused mainly on human or animal endothelial cells of kidney, retina, myocardium, and human umbilical vein endothelial cells (HUVECs). Thanks to these studies, the mechanisms by which hyperglycemia initiates apoptosis are better understood. These mechanisms include oxidative stress, increased intracellular Ca^2+^, mitochondrial dysfunction otherwise known as the mitochondria apoptosis pathway, changes in intracellular fatty acid metabolism, activation of Mitogen activated protein kinases (MAPK) signaling pathways, and impaired phosphorylation activation of the protein kinase Akt [24, 31] (Figure 4).

One specific study with HUVECs demonstrated that elevated glucose induces apoptosis and downregulates VEGF in HUVECs by inhibiting p42/44 MAP kinase activation. High glucose also significantly increased Bax protein but did not affect Bcl-2, thereby elevating the Bax/Bcl-2 ratio which activates cleavage of procaspase 3 into active caspase-3, in turn triggering apoptosis in HUVECs. When VEGF was added to the HUVECs exposed to high glucose, apoptosis was prevented through inhibition of elevated ROS generation, calcium overload and activation of the mitochondria apoptosis pathway. VEGF significantly decreased Bax expression without affecting the Bcl-2 level and attenuated the increase in caspase 3 activity. VEGF in HUVECs could also decrease H_2_O_2_ production at 48 hours high glucose stimulation, suggesting that it inhibits the ROS/NF-*κ*B/JNK/Caspase-3 pathway [24]. 

One earlier study with human aortic endothelial cells and bovine aortic endothelial cells exposed to high D-glucose also showed a significant increase in the Bax/Bcl-2 ratio followed by an increase in caspase-3 activity and cell death. They proved that Bax inserts the mitochondrial membranes, triggering a transformation of mitochondrial function after high D-glucose treatment of the human aortic endothelial cells. This study also demonstrated that high D-glucose leads to phosphorylation of p38 Mitogen-Activated Protein Kinase (p38 MAPK) mediated by MEK-kinase1 (MEKK1) downstream of bax-caspase proteases and thereby causes apoptosis of aortic endothelial cells [27]. 

Another study with HUVECs also investigated the role of the three MAPK pathways: the extra cellular signal-regulated kinases (ERK), the c-Jun NH 2-Terminal Kinase /stress-activated protein kinases (JNK/SAPK), and p38 MAPK. They found that high glucose triggers apoptosis via ROS through activating JNK/SAPK. This study showed no significant role for the other two MAPK pathways [95]. Later in 2005 this research group found that hyperglycemia induces ROS generation through a PI3K-dependent pathway. They observed that hyperglycemia causes a PI3K/Akt-dependent upregulation of Cyclooxygenase 2 (COX-2) expression and thereby an increase of prostaglandin E2 (PGE2) production and subsequently a caspase-3 activation and facilitation of apoptosis in HUVECs. These findings were supported by the fact that LY294002 or wortmann (both PI3K/Akt inhibitors) prevented the COX-2 mediated PGE2 production and subsequently the caspase-3 activity, and apoptosis. Inhibition of COX-2 with a selective COX-2 inhibitor NS398 also inhibited PGE2 production, caspase-3 activity and apoptosis in HUVECs treated with high glucose levels. Moreover they found that hyperglycemia could trigger NF-*κ*B activation and that dominant-negative IkB*α* could prevent COX-2 expression and apoptosis, implying that NF-*κ*B activation can lead to COX-2 mediated PGE2 production and apoptosis in HUVECs exposed to hyperglycemia [96]. 

There are several studies with HUVECs that prove that high glucose-induced apoptosis is associated with an increase in Ca^2+^ current, resulting from Ca^2+^ entry mediated by store-operated channels. An increased amount of cellular Ca^2+^ causes more mitochondrial Ca^2+^ uptake. Ca^2+^ accumulation in mitochondria is one of the primary causes for mitochondrial permeability transition, through the opening of the PT-pore and this is an important key factor in the apoptotic pathway [24].

The involvement of the intracellular fatty acid metabolism is suggested by a study in which HUVECs were treated with high glucose concentrations for 24 hours and showed inhibition of fatty acid oxidation, increases in fatty acid esterification and the concentration of malonyl-CoA before apoptosis was induced. This finding suggests a causal relation of alterations in intracellular fatty acid and apoptosis in hyperglycemia. Decreases in mitochondrial membrane potential and cellular ATP content also preceded apoptosis. All these metabolic alterations are associated with an increase in caspase-3 activity and an impaired ability of insulin at a physiological concentration to activate Akt. Finally an antiapoptotic role for AMPK is suggested in this study because incubation of the HUVECs with 5-aminoimidazole-4-carboxamide-riboside (AICAR), an AMPK activator, prevented all of the above changes. Likewise, a similar decrease in caspase-3 activity was observed when AMPK activity was increased by infecting HUVEC with constitutively active AMPK using an adenoviral vector [33]. 

Recently, a study with human pancreatic islet microvascular endothelial cells (MECs) proved that sustained hyperglycemia progressively affects cellular survival and proliferation and increases apoptosis of cultured MECs. After 24 to 48 hours, apoptosis was detected in high glucose both by DNA fragmentation and activation of the caspase family. In this study they found that the islet MECs, under conditions of sustained hyperglycemia, showed a progressively reduced phosphorylation of Akt, suggesting an interference with the pathways involved in Akt activation. Hyperglycemia also downregulated the tyrosine phosphorylated form of the transmembrane protein nephrin. It is known that phosphorylated nephrin associates with PI3K and activates the multifunctional Akt-dependent pathways. This suggests that hyperglycemia-induced apoptosis of islet endothelium likely involves the nephrin-mediated signaling cascade, wherein phosphorylation of the tyrosine sites within the intracytoplasmic C terminal domain of nephrin activates mitogen-activated protein kinase p38 and JNK and thereby the transcription factor activating protein-1 (AP-1)/c-Jun, which modulates apoptosis. The study with islet MECs also detected an increased production of the proinflammatory cytokine IL-1*β*, which can induce Fas expression enabling Fas-mediated apoptosis [31]. 

## 8. Conclusion

The relation between diabetic micro- and macroangiopathy and endothelial dysfunction is complex and is still a subject of extensive research. Especially in type 2 diabetes a lot of factors are involved including hyperglycemia, hyperinsulinemia, insulin resistance, dyslipidemia, hypertension, and obesity, which all influence each other and probably intensify each others actions. More insights into the exact mechanisms underlying endothelial dysfunction may lead to important treatment strategies which can significantly reduce the morbidity and mortality rate caused by endothelial dysfunction especially in diabetes patients. Although apoptosis is a natural phenomenon in all multicellular organisms, an increased and accelerated rate of apoptosis of endothelial cells is probably a crucial factor in diabetic co-morbidity. There are many pathways involved in activating endothelial cell apoptosis and all of these pathways can be activated in multiple ways. A common mechanism causing endothelial dysfunction and endothelial cell apoptosis is oxidative stress. Several studies show contradictory results regarding a possible role for antioxidants in the treatment to prevent micro- and macroangiopathy. However a treatment aimed at reducing oxidative stress in endothelial cells may be an answer to this major problem, especially since diabetes will soon become an even bigger health problem involving more than 5% of the world population.

## Abbreviations

ACE: Angiotensin Converting Enzyme
AGE: Advanced Glycation End-products
AIF: Apoptosis Inducing Factor
ANG-II: Angiotensin II
AMP: Adenosine Monophosphate
AMPK: AMP-activated Protein Kinases
APAF-1: Apoptotic Peptidase Activating Factor 1
ARB: Angiotensin II type 1 Receptor Blocker
ASK-1: Apoptosis Signal Regulating Kinase 1
BMI: Body Mass Index
cGMP: Cyclic Guanosine MonoPhosphate
COX: Cyclooxygenase
CRP: C-Reactive Protein
CVD: Cardio Vascular Disease
DISC: Death Inducing Signaling Complex
DNA: Deoxyribonucleic Acid
Egr-1: Early Growth Response Protein 1
EMP: Endothelial Micro Particle
ERK: Extra cellular signal-Regulated Kinase
ET-1: Endothelin-1
FADD: Fas Associated Death Domain
FasL: Fas-ligand or CD95-ligand
FFA: Free Fatty Acids
FOXO-1: Forkhead box O1
GLUT-4: Glucose Transporter-4
GADP: Glyceraldehyde 3-Phosphate
GSH: Gluthathione
HbA1c: Hemoglobin A1c
HDL: High Density Lipoproteins
HUVEC: Human Umbilical Vein Endothelial Cell
ICAD: Inactive Caspase Activated DNase
ICAM: Intracellular Adhesion Molecule
IL-6: Interleukine-6
IR: Insulin Receptor
IRS-1: Insulin Receptor-Substrate-1
IGT: Impaired Glucose Tolerance
JNK: c-Jun NH 2-Terminal Kinase
LAM: Leucocyte Adhesion Molecule
MAPK: Mitogen-Activated Protein Kinase
MCP-1: Monocyte Chemotactic Protein-1
MEC: Microvascular Endothelial Cell
MEKK1: Methyl Ethyl Ketone Kinase-1
MP: Micro Particle
mRNA: messenger Ribonucleic Acid
NADP(H): Nicotinamide adenine dinucleotide phosphate
NEFA: Non-Esterified Fatty Acid
NO: Nitric Oxide
NOS: Nitric Oxide Synthase
NF-*κ*B: Nuclear Factor *κ*B
OPG: Osteoprogesterin
PARP: Poly ADP-Ribose Polymerase
PAI-1: Plasminogen Activator Inhibitor-1
PMN: Polymorphonuclear Leukocyte
PTP: Permeability Transition Pore
PDGF: Platelet-Derived Growth Factor
PGE2: Prostaglandin E2
PGI2: Prostacyclin
PI-3K: Phosphoinositide-3 Kinase
ROS: Reactive Oxygen Species
SA*β*-gal: *β*-Galactosidase
SAPK: Stress-Activated Protein Kinases
SOD: Superoxide Dismutase
STZ: Streptozotocin
T1DM: Type 1 Diabetes Mellitus
T2DM: Type 2 Diabetes Mellitus
TNF*α*: Tumor Necrosis Factor-*α*
t-PA: Tissue-type Plasminogen Activator
TRAIL: TNF-Related apoptosis Inducing Ligand
VCAM: Vascular Cell Adhesion Molecule
VEGF: Vascular Endothelial Growth Factor
VLDL: Very Low Density Lipoproteins
VSMC/VSM: Vascular Smooth Muscle (Cell)
vWF: Von Willebrand Factor.
